# Long‐Living Holes in Grey Anatase TiO_2_ Enable Noble‐Metal‐Free and Sacrificial‐Agent‐Free Water Splitting

**DOI:** 10.1002/cssc.202001045

**Published:** 2020-08-14

**Authors:** Ning Liu, Shiva Mohajernia, Nhat Truong Nguyen, Seyedsina Hejazi, Fabian Plass, Axel Kahnt, Tadahiro Yokosawa, Andres Osvet, Erdmann Spiecker, Dirk M. Guldi, Patrik Schmuki

**Affiliations:** ^1^ Department of Materials Science (WW4) LKO University of Erlangen-Nuremberg Martensstrasse 7 91058 Erlangen Germany; ^2^ Department of Chemistry and Pharmacy Interdisciplinary Center for Molecular Materials (ICMM) University of Erlangen-Nuremberg Egerlandstr. 3 91058 Erlangen Germany; ^3^ Leibniz Institute of Surface Engineering (IOM) Permoserstr. 15 04318 Leipzig Germany; ^4^ Institute of Micro- and Nanostructure Research (WW9) & Center for Nanoanalysis and Electron Microscopy (CENEM) University of Erlangen-Nuremberg Cauerstrasse 6 91058 Erlangen Germany; ^5^ Department of Materials Sciences 6 i-MEET University of Erlangen-Nuremberg Martensstrasse 7 91058 Erlangen Germany

**Keywords:** long-living holes, noble-metal-free, sacrificial-agent-free, titanium dioxide, water splitting

## Abstract

Titanium dioxide has been the benchmark semiconductor in photocatalysis for more than 40 years. Full water splitting, that is, decomposing water into H_2_ and O_2_ in stoichiometric amounts and with an acceptable activity, still remains a challenge, even when TiO_2_‐based photocatalysts are used in combination with noble‐metal co‐catalysts. The bottleneck of anatase‐type TiO_2_ remains the water oxidation, that is, the hole transfer reaction from pristine anatase to the aqueous environment. In this work, we report that “grey” (defect engineered) anatase can provide a drastically enhanced lifetime of photogenerated holes, which, in turn, enables an efficient oxidation reaction of water to peroxide via a two‐electron pathway. As a result, a Ni@grey anatase TiO_2_ catalyst can be constructed with an impressive performance in terms of photocatalytic splitting of neutral water into H_2_ and a stoichiometric amount of H_2_O_2_ without the need of any noble metals or sacrificial agents. The finding of long hole lifetimes in grey anatase opens up a wide spectrum of further photocatalytic applications of this material.

## Graphical abstract

**Long‐living photogenerated holes**: Grey anatase, in comparison to white anatase, provides a drastically enhanced lifetime for trapped photogenerated holes. This allows to construct a Ni@grey anatase TiO_2_ catalyst that can photocatalytically split neutral water into H_2_ and a stoichiometric amount of H_2_O_2_ without the need of any noble metals or sacrificial agents.Institute and/or researcher Twitter usernames: @FAU_Germany

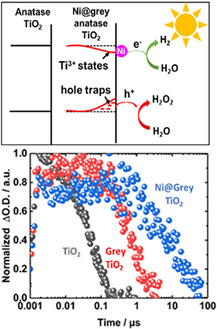

Direct water splitting by means of semiconductor‐based photocatalysts constitutes one of the most intriguing routes to produce hydrogen using solar energy.^1^, ^2^ A large number of semiconductors with suitable energies of their band edges exist, but only a few of them photo‐catalyze the overall water splitting in the absence of any sacrificial agents.^3^, ^4^, ^5^, ^6^, ^7^ Classic examples are binary oxides such as K_4_Nb_6_O_17_, KTaO_3_, or SrTiO_3,_ which have been reported to split pure water or aqueous alkaline solution into H_2_ and O_2_ ‐ however, they typically require the use of a noble metal co‐catalyst such as Pt or Rh.^3^, ^4^, ^5^, ^6^, ^7^ More recent examples are noble metal loaded sulfide‐ and nitride‐based semiconductors, namely carbon‐nitrides loaded with CoP and Pt,^8^ or Pt and RuO_2_.^8^, ^9^, ^10^, ^11^

In spite of these findings, titanium dioxide (TiO_2_), in its anatase form, remains the most investigated semiconductor for photocatalysis, due to its exceptional photocorrosion resistance, favorable economics and energetics.^12^, ^13^ However, overall water splitting, i. e. the decomposition of water into H_2_ and O_2_ in stoichiometric amounts, has hardly ever been realized,^1^, ^6^ and these very few examples report only on very moderate efficiencies.^14^, ^15^ Almost exclusively, investigations on anatase focus on H_2_ generation using sacrificial agents (such as methanol or ethanol) to capture the photogenerated holes – this in turn enables photocatalytic H_2_ generation with reasonable efficiencies.^16^, ^17^, ^18^

For titania, in pure water, the extremely slow kinetics of charge‐transfer processes across the anatase/water‐interface is the major obstacle to high H_2_ production rates. Both the reductive electron‐transfer to form H_2_ and even more the oxidative hole‐transfer to oxidize water are sluggish, despite the fact that both reactions are thermodynamically feasible.

In terms of electron extraction, the common approach is to use noble metal co‐catalysts, such as Pt, Pd or Rh as a decoration on the titania semiconductor; they act as electron sinks and facilitate efficient electron‐transfer across the interface. High costs for noble metals turned the attention to earth‐abundant alternatives with a low overpotential for the H_2_ evolution.^3^, ^4^, ^5^, ^6^, ^7^, ^12^, ^13^ To this end, nickel is one of the most promising candidates for proton reduction in combination with various semiconductors. Nickel acts as efficient co‐catalyst for the hydrogen generation in combination with, for example, CdS, if the hole‐transfer pathway is provided by sacrificial agents.^19^, ^20^, ^21^ The most prominent example of the use of Ni is as a decoration on perovskite SrTiO_3_. In this example hole transfer to H_2_O is possible due to the catalyzing properties of the SrTiO_3_ surface for the oxygen evolution reaction (OER) and an additional beneficial effect of Ni‐oxides.^22^, ^23^ However, anatase shows a drastically lower activity to catalyze the oxygen evolution reaction.^24^, ^25^, ^26^, ^27^ As a result, on Ni‐decorated anatase surfaces sacrificial agents are mandatory to allow for a reasonable hole transfer.^28^, ^29^

In this work, we document for the first time that using so‐called grey anatase (a specific, defect‐engineered modification of hydrogen‐reduced anatase as described in the Supporting Information and in detail in Refs. [30–32]) can provide, due to a high density of deep trapping states, extremely long‐living photogenerated holes and thus opens up an effective hole‐transfer pathway that allows an efficient oxidation of pure neutral water. As a consequence, also the electron transfer can be mediated with a stable Ni co‐catalyst as outlined below. This Ni@grey anatase TiO_2_ catalyst is cost effective and highly efficient: The photoreaction occurs without noble metals and without any sacrificial agent. The catalyst enables photoexcited holes to surface‐oxidize water to peroxide on one hand and allows Ni as a means for electron transfer to activate the catalyst for H_2_ evolution on the other hand. The basic processes are depicted in Figure 1a. To obtain the Ni@grey anatase TiO_2_ catalyst, we loaded Ni(OH)_2_ onto commercial anatase nanoparticles by a solution impregnation method, followed by hydrogenation at 500 °C in a H_2_ atmosphere – this hydrogenation step leads to a reduction of Ni species and concomitantly forms the desired reduced grey anatase. For reference, we also included the widely investigated black form of anatase^33^ (see Figure S1 on synthesis and characteristics of “white”, “grey”, and “black” titania in the Supporting Information and Experimental Section). It is worth nothing that the formation of grey anatase by thermal H_2_ reduction was studied on various titania nanostructures namely TiO_2_ nanotubes and in presence of sacrificial agents showed a strongly enhanced photocatalytic and photoelectrocatalytic performance in comparison to pristine anatase.^34^, ^35^

**Figure 1:**
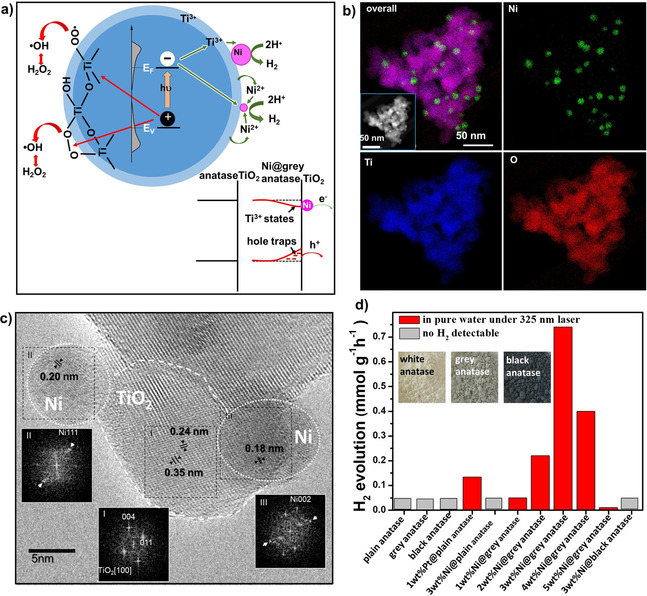
a) Photocatalytic generation of H_2_ and H_2_O_2_ on Ni@grey anatase TiO_2_ under illumination. Light absorption by Ni@grey anatase TiO_2_ generates electrons and holes; electrons travel to the Ni‐decorated surface and lead to proton reduction, holes are trapped on deep states (become long living) and, thus, enable water oxidation to H_2_O_2_. Scanning TEM: b) elemental EDX mapping and c) corresponding HR‐TEM image of Ni@grey anatase TiO_2_. Indicated are interplanar spacing as well as corresponding FFT patterns corresponding to anatase and Ni^0^. d) Photocatalytic H_2_ production from Ni@grey anatase TiO_2_ in pure neutral water under UV illumination (325 nm, 50 mW) compared with plain anatase TiO_2_, grey anatase TiO_2_, Ni@plain anatase TiO_2_, and Pt@plain anatase TiO_2_.

A crucial difference from white (plain) anatase is that “grey” and “black” anatase show a variation of the density of states distribution (DOS) at the conduction and valence band edge depending on the level of reduction of anatase.^30^, ^36^ To synthesize an optimum Ni‐decorated catalyst, parameter screening experiments were performed, in which the level of Ni decoration and the hydrogenation conditions were systematically altered (see Table S1). For the optimized catalyst, energy dispersive spectroscopy mapping of the transmission electron microscope (TEM‐EDS) (Figure 1b gives the elemental distribution for Ni, O, and Ti) shows Ni to be evenly distributed as ca. 10 nm sized particles on the grey TiO_2_ surface (see also Figure S3). HRTEM images (Figure 1c) reveal a lattice spacing of 0.35 nm for the grey TiO_2_ substrate, which correlates well with the (101) lattice planes of anatase and grey anatase. For the Ni particle, the d‐spacing is 0.21 nm and the Fourier transformation results are in line with a (200) *d*‐spacing of metallic nickel (Figure 1c). Optimized hydrogenation conditions are established for a 1 h H_2_ treatment at 500 °C which leads to the most efficient photocatalyst for water splitting (Figure 1d). Higher hydrogenation temperatures of, for example, 600 or 700 °C produce darker forms (including “black” titania). However, these darker forms are less photocatalytically active, see Figure 1d.^33^, ^37^ An optimized loading with Ni was observed for a nominal 3 % loading (see Figure 1d).

This Ni@grey anatase TiO_2_ was examined as water splitting photocatalyst without the addition of any sacrificial reagents. When using UV light irradiation (HeCd laser, 325 nm, 50 mW) we note a substantial photocatalytic production of hydrogen with an H_2_ evolution rate of ∼700 μmol h^−^ g^−1^ from neutral water that correlates to a quantum efficiency of 2.3 % (see also Supporting Information Figure S2).

We then investigated the water oxidation products. In fact, no O_2_ evolution could be detected as a photocatalytic hole transfer product from Ni@grey anatase, but clearly the formation of H_2_O_2_ could be identified first qualitatively (using simple H_2_O_2_ test stripe (Sensafe Co.) or horseradish peroxidase (HRP)‐based testing^38^ – see inset of Figure 2a and details in the Supporting Information). The quantitative H_2_O_2_ yield over illumination time can be determined by KMnO_4_ titration^39^, ^40^ (see Figure 2a). As H_2_O_2_ is often reported to be prone to illumination or even to serve as a charge carrier capture agent,^41^ we carried out various reference tests and confirmed that under our experimental conditions neither a light induced decay nor a photocatalytic loss^42^ of H_2_O_2_ takes place (see Figures S4 and S5).

**Figure 2:**
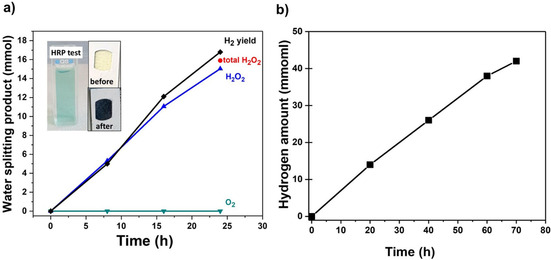
a) H_2_ evolution and water oxidation products. Identification of hole transfer reaction products from qualitative (commercial H_2_O_2_ test stripes or HRP‐based tests of H_2_O_2_ tests) and quantitative derivation over time for Ni@grey anatase TiO_2_ splitting pure neutral water under UV irradiation. H_2_O_2_ was determined from KMnO_4_ titration as described in the Supporting Information. b) Time dependence of photocatalytic H_2_ for long‐term experiment using Ni@grey anatase TiO_2_ in pure neutral water.

As a result, over the entire photocatalytic reaction time, a total amount of H_2_O_2_ is produced that leads to a H_2_/H_2_O_2_ ratio close to a 1 : 1 stoichiometry (Figure 2a). In long‐term experiments (Figure 2b), during which photocatalytic experiments were repeated in 24 h intervals, the Ni@grey anatase TiO_2_ features a high stability over a time period of more than 70 h. Importantly, for Ni@plain anatase or Ni@black anatase no appreciable photocatalytic formation of H_2_O_2_ over 6 h of illumination could be observed (see data in Figure S5). Therefore, the unique water splitting performance observed here must be ascribed to the features of grey titania. It also should be noted that based on X‐ray photoelectron spectroscopy (XPS) result and selected area diffraction (SAED) pattern analysis of the extracted powder after illumination, no significant changes are observed neither in the crystallographic phases nor in the chemistry of the powder (see Supporting Information Figure S8). This is also in line with literature data using electron paramagnetic resonance (EPR) that show the defect signatures in grey anatase after catalysis to be virtually the same as the one observed for the initial grey material.^43^

In order to elucidate the origin of this astonishing feature of grey anatase, we investigated the charge carrier dynamics by in situ femtosecond and nanosecond transient absorption measurements. An overview of the transient absorption spectra and the absorption time profiles for grey titania and Ni@grey titania is compiled in Figure 3 (additional data are given in Figure S6 and S7). An evaluation of the data in Figure 3 for white anatase TiO_2_, grey anatase, and Ni@grey anatase yields free electron lifetimes for all materials in the range of picoseconds (Tables S2 and S3 in the Supporting Information), which is in sound agreement with general literature findings.^23^ However, for holes, the lifetimes of the different anatase forms are drastically different—they range from nano‐ to microseconds when comparing white with grey material (Figure 3). Two types of hole traps, namely shallow traps with an absorption in the range from 400 to 450 nm and deep traps with an absorption in the 500 to 700 nm range, are well established for anatase TiO_2_.^44^ For the shallow‐trapped holes, the corresponding lifetimes are 6 ns for anatase TiO_2_, 14 ns for grey anatase TiO_2_ and 3200 ns for Ni@grey anatase TiO_2_ (Table S3). For the deep‐trapped holes lifetimes show even more pronounced differences; relative to plain anatase TiO_2_, the deep‐trapped hole lifetime in grey anatase increases from 64 to 820 ns and for Ni@grey anatase TiO_2_ even to a value of 14.2 μs (Table S3).

**Figure 3:**
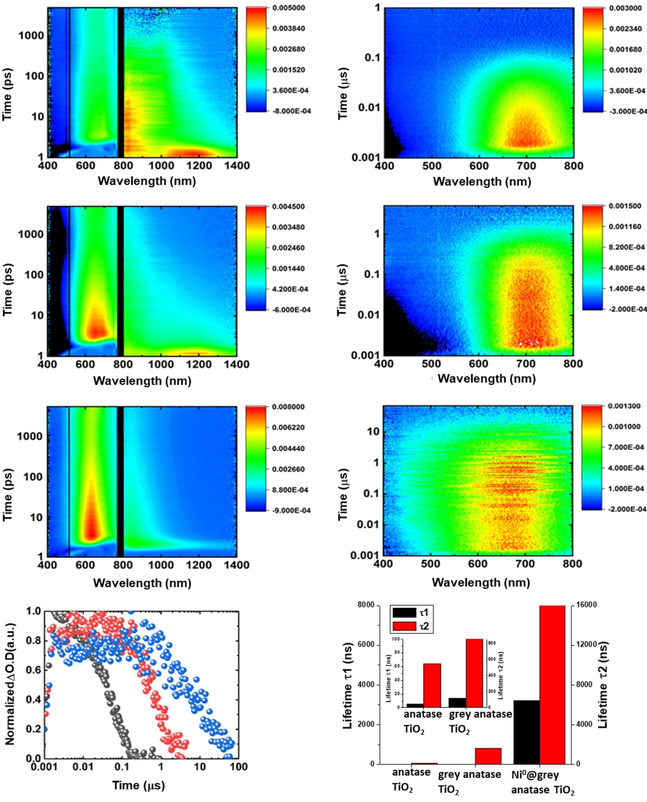
Femtosecond (left) and nanosecond (right) transient absorption spectra obtained upon laser photolysis (258 nm) of anatase TiO_2_, grey anatase TiO_2_, Ni@grey anatase TiO_2_ (from the top to the bottom) in argon‐saturated aqueous suspensions. The lower left image depicts the time absorption profiles at 650 nm for anatase TiO_2_ (black), grey anatase TiO_2_ (red) and Ni@grey anatase TiO_2_ (blue) corresponding to deep‐trapped holes. The lower right image shows a histogram of the lifetimes of the shallow‐trapped holes (*τ*_1_‐black) and deep‐trapped holes (*τ*_2_‐red) obtained from the corresponding nanosecond transient absorption measurements.

The existence of a high density of deep trapping states for holes in dark anatase TiO_2_ is in line with recent literature on the electronic structure of hydrogenated TiO_2_, as outlined in Figure S1. An optimized level of reduction affects not only the conduction band and allows facilitated electron transfer^12^, ^31^, ^33^, ^34^, ^45^ but importantly also valence band spectra of white, grey and black TiO_2_ show a very different valence state distribution (or deep traps) tailing into the bandgap of TiO_2_.^30^, ^31^, ^32^, ^46^

For the grey anatase sample, in literature, the valence band maximum energy is blue shifted by approximately 0.5 to 0.7 eV towards the vacuum level compared with the typical valence band of anatase TiO_2_.^32^, ^47^ Such a shift in the density of states (DOS) in grey anatase TiO_2_ is in line with DFT calculations for hydrogenated material.^30^, ^47^ Please note that for fully black anatase the shift is even larger and extends to 1.5‐1.6 eV in line with literature^47^).

Corresponding alterations of the electronic structure in grey titania are also evident from PL measurements. Figure 4a shows the PL spectra of anatase TiO_2_ and grey anatase (see the Supporting Information for experimental details). A strong PL signal, which is centered at around 520–530 nm, is observed for grey TiO_2_, while this PL is weak in plain anatase TiO_2_. The origin of this PL peak in anatase has been investigated in depth by Pallotti et al.^48^ – t can be attributed to a recombination of conduction band electrons with deep‐trapped holes that lie approx. 2.3 eV below the conduction band of anatase TiO_2_.^48^, ^49^ This finding fully supports the notion of a valence band tailing (or deep trapping states) in grey titania. In energy these hole trapping states lie at an energetic optimum for holes to be transferred to water; leading to water oxidation, to H_2_O_2_ (Figure 4b). For Ni@grey anatase, the rapid transfer of electrons to the Ni‐cocatalyst eliminates emissive recombinations of valence band carriers with deep‐trapped holes and, in turn, causes a quantitative PL quenching.

**Figure 4:**
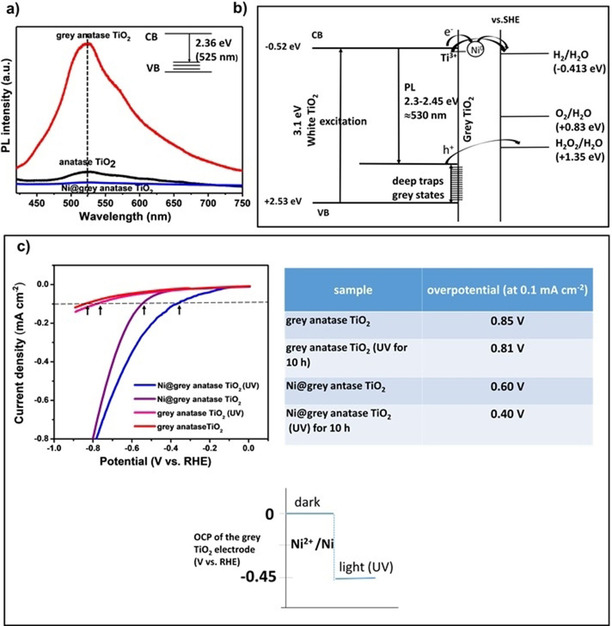
a) Photoluminescence spectra of anatase TiO_2_ and grey anatase TiO_2_ excited by a 325 nm laser. The peak 525 nm corresponds to a recombination of electrons with deep‐trapped holes as illustrated in the inset and in line with Ref. [32]. b) Schematic of energetic levels involved in photocatalytic process of grey titania and relative position of deep trapping states to the redox potential of water (pH 7). c) Cathodic linear sweep voltammograms curves of the samples in the dark: grey, Ni@grey TiO_2_, and in Ar‐saturated water solution (0.1 M Na_2_SO_4_), showing the electrocatalytic effect of Ni on Ni@grey anatase TiO_2_ for H_2_ evolution.

To probe the Ni involvement in the water splitting reaction, cathodic electrochemical voltage scans were conducted with films of grey anatase and Ni@grey anatase (see Figure 4c). Most importantly, the presence of the Ni‐cocatalyst lowers the overpotential (*η* at −0.1 mA cm^−2^) for H_2_ evolution from *η*=0.85 V vs. RHE for grey anatase TiO_2_ to *η*=0.60 V vs. RHE for Ni@grey anatase TiO_2_. It is noteworthy that after 10 h of UV illumination under open‐circuit potential (OCP), the overpotential for Ni@grey anatase TiO_2_ is even further reduced to *η*=0.45 V vs. RHE. This not only shows the effect of Ni as H_2_ evolution catalyst but also the high stability of the metallic Ni‐cocatalyst under illumination. The OCP potential of the electrode under illumination was measured to be at 0.45 V RHE, i. e. under operating conditions the electrode is in potential range where Ni(metal) can effectively catalyze H_2_ evolution, see Figure 4c.

Overall, the present results show clearly that water splitting by the Ni@grey anatase TiO_2_ catalyst proceeds via the two‐charge pathway to form H_2_ and H_2_O_2_ rather than the four‐charge pathway to afford H_2_ and O_2_. Considering that for Ni@plain anatase or Ni@black anatase no appreciable photocatalytic formation of H_2_O_2_ over extended times of illumination could be observed, the unique characteristics of the catalyst introduced here must be ascribed to grey titania and its optimum hole‐trapping ability and particularly the provision of sufficiently long life‐times of trapped holes to enable reaction with water to H_2_O_2_.

Most importantly, the photocatalytic overall stoichiometric water splitting on Ni@grey anatase proceeds without a noble metal co‐catalyst and without any sacrificial agent. The H_2_ evolution efficiency of Ni@grey anatase reaches ∼700 μmol h^−1^ g^−1^ from neutral water, which is higher than any H_2_ evolution rate reported for a TiO_2_‐based catalyst for water splitting in pure water.^14^, ^15^ Moreover, Ni@grey anatase is stable – the high rate of hydrogen evolution from pure water is maintained over a period of several 100 h. The work, moreover, highlights that one of the most important features of grey anatase is the establishment of extremely long lifetimes for light generated holes.

## Experimental Section

### Catalyst preparation

We used commercial anatase nanopowder (purity: 99.8 %, particle size: 25–35 nm, Aldrich) as a precursor for decoration and hydrogenation. A series of Ni@TiO_2_ samples with nominal Ni loadings of 0–5 wt% were initially prepared by a solution impregnation method. For this, nickel(II) nitrate hexahydrate and glycerol (1 : 2 molar ratio) were added to DI water (200 mL) to form an aqueous nickel(II)−glycerol complex. The exact masses of nickel(II) nitrate hexahydrate and glycerol used depended on the target nominal Ni loading. Then, anatase (1 g) was added to the Ni‐complex solution under continuous stirring, and the nickel−glycerol complex was then precipitated on the TiO_2_ support by a dropwise addition of 0.5 M NaOH until a pH of 12 was reached. The resulting suspension was stirred for further 1 h, then the resulting light green powder (presumably Ni(OH)_2_@TiO_2_) was collected by vacuum filtration.

For H_2_ treatments, different Ni loaded TiO_2_ powders were annealed in hydrogen atmosphere (purity 99.999 %, Linde) at different temperatures (400, 500, 600, 700 °C) for 1 h. These samples show color changes (involving “grey” and “black”, see Figure S1). After the hydrogenation, these samples (i. e. ‘fresh’ samples) were then directly (under Ar atmosphere) transferred, for example, for photocatalytic or electrochemical measurements, to deaerated (Ar bubbled) DI water (see section “Photocatalytic and electrochemical properties”).

### Photocatalytic and electrochemical properties

Photocatalytic hydrogen generation was measured under open circuit conditions from pure neutral DI water using a UV laser (HeCd laser, Kimmon, Japan; *λ*=325 nm, 55 mW cm^−2^). The amount of H_2_ produced was measured using a Varian gas chromatograph with a TCD detector. To prepare suspensions for H_2_ measurements, 2 mg TiO_2_ powders (except for Figure S2) were dispersed in 10 mL of DI water (18 MΩ cm) with ultrasonication for 30 min. During illumination, the suspensions were continuously stirred. To test the stability of catalyst for hydrogen evolution, repeated cycle measurements of hydrogen evolution as photocatalyst over a testing period of 5 days (measured in intervals of 24 h) were carried out. The powder remained the same for each cycle.

The linear sweep voltammetry experiments were conducted on glassy carbon electrodes. From the cathodic scans with samples, which were directly taken after preparation, and which were illuminated for 10 h (365 nm LED, 100 mW), we obtain different H_2_ evolution characteristics and the overpotential taken at a proton reduction current of 0.1 mA cm^−2^.

H_2_O_2_ was qualitatively detected using commercial peroxide check stripes 481015 (Sensafe Co. USA). For each measurement one test stripe was dipped into the solution with constant, gentle back and forth motion for 5 s. Then the test stripe was removed and shaken to remove the excess water. After waiting for 15 s, the color was matched to the color chart provided by the company. Additional confirmation for H_2_O_2_ detection was obtained using the ABTS‐HRP method.^38^, ^40^ For this, a solution containing horseradish peroxidase (HRP RZ=3.2), 2,2′‐azino‐bis (3‐ethylbenzthiazoline‐6‐sulfonic acid), diammonium salt (ABTS) and phosphate buffered saline (PBS) was used. The spectrophotometric identification was performed as follows: 3 mL of the solution HRP‐ABTS PBS was added in the sample cuvette following by addition of 0.1 mL of the suspension right after illumination. Quantitative determination of H_2_O_2_ was carried out by titration with KMnO_4_.^39^ For this, 10 mL of the slurry was mixed with 10 mL of concentrated (4 M) H_2_SO_4_. The KMnO_4_ was added dropwise to the stirred solution until a permanent pink color was apparent. The procedure was verified using standard H_2_O_2_ solutions (prepared from H_2_O_2_ 30 wt% Merck).^39^ These experiments (Figures S4, S5) were carried out, as it is well‐known that various peroxo−titania complexes can be generated on TiO_2_ surfaces.^27^, ^50^, ^51^, ^52^ Also experiments were carried out to rule out artefacts such as a light induced decomposition of H_2_O_2_.^53^, ^54^, ^55^

Carrier life‐time measurements were carried out using femtosecond and nanosecond laser photolysis transient absorption spectroscopy. Femtosecond transient absorption measurements were performed with output from a Ti:sapphire laser system (CPA2110, Clark – MXR Inc.): 775 nm, 1 kHz, and 150 fs FWHM pulses. The excitation wavelength was generated by third harmonic generation (258 nm), pulse widths of <150 fs and energies of 400 nJ per pulse were selected. The transient absorption detection was performed with a transient absorption pump/probe system (TAPPS, Ultrafast Systems). Nanosecond transient absorption experiments were performed with an EOS spectrometer (Ultrafast Systems LLC). The pump pulses at *λ*=258 nm came from the Ti:sapphire laser system described above. The probe pulse (2 kHz, 0.5 ns pulse width), which was generated in a photonic fiber, was synchronized with the femtosecond amplifier. Both measurements were carried out in‐situ using 2 mm quartz cuvettes filled with the suspensions of interest (e. g., anatase powders in pure neutral water).

## Conflict of interest

The authors declare no conflict of interest.

## Supporting information

As a service to our authors and readers, this journal provides supporting information supplied by the authors. Such materials are peer reviewed and may be re‐organized for online delivery, but are not copy‐edited or typeset. Technical support issues arising from supporting information (other than missing files) should be addressed to the authors.

Supplementary
