# Supplementary material for: Long‐Living Holes in Grey Anatase TiO2 Enable Noble‐Metal‐Free and Sacrificial‐Agent‐Free Water Splitting
Source: ChemSusChem. 2020 Aug 14;13(18):4937–44. doi: 10.1002/cssc.202001045 (PMC7540354; doi:10.1002/cssc.202001045)
Supplement: Supplementary file 1 — Supplementary [file CSSC-13-4937-s001.pdf]

ChemSusChem

Supporting Information

Long-Living Holes in Grey Anatase TiO_2 Enable Noble-Metal-Free and Sacrificial-Agent-Free Water Splitting

Ning Liu[+], Shiva Mohajernia[+], Nhat Truong Nguyen, Seyedsina Hejazi, Fabian Plass, Axel Kahnt, Tadahiro Yokosawa, Andres Osvet, Erdmann Spiecker, Dirk M. Guldi, and Patrik Schmuki*© 2020 The Authors. Published by Wiley-VCH GmbH. This is an open access article under the terms of the Creative Commons Attribution License, which permits use, distribution and reproduction in any medium, provided the original work is properly cited.This publication is part of a Special Collection highlighting "The Latest Research from our Board Members". Please visit the Special Collection at https://bit.ly/cscBoardMembers

Characterization of the samples:

High-resolution transmission electron microscopy (HRTEM) was performed using an image-side aberration corrected FEI Titan^3 80-300 transmission electron microscope (TEM) operated at an acceleration voltage of 200 kV. Selected area electron diffraction (SAED) patterns were acquired with a Philips CM 300 UT TEM at an acceleration voltage of 300 kV. For TEM, the powder samples were diluted with deionized water. After 3 minutes of ultrasonication, the dispersions were drop-casted onto TEM copper grids coated with a lacey carbon film and dried overnight before investigation.

For morphological characterization of the samples, we used a field-emission scanning electron microscope (FE-SEM, Hitachi SEM FE 4800).

Information on the chemical composition of the powders was obtained from X-ray photoelectron spectroscopy (XPS, PHI 5600 XPS spectrometer). XPS spectra were acquired using an Al standard X-ray source with a pass energy of 23.5eV. All XPS element peaks were shifted to the C1s position of 284.8 eV.

The photoluminescence (PL) of the epitaxial samples was excited with a 325 nm He-Cd laser and the spectra were recorded in Ar atmosphere at room temperature with an iHR320 monochromator and a Synergy Si CCD camera (both Horiba Jobin-Yvon). The spectra were corrected for the spectral sensitivity of the setup, determined with the help of a calibrated halogen lamp.

The reflectance of the samples was measured using UV/Vis spectrometer (Lambda 950) in a range of 800–200 nm using an integrating sphere.

Table S1: Various preparation parameters of the photocatalyst and the resulting H_2 evolution rates in pure water under 325 nm UV illumination

No.	Ni loading (wt%)	Hydrogenation condition (°C for 1 h)	H_2 evolution rate (μmol x $h^{-1}g^{-1}$)
1	0	no	0
2	0	500	0
3	0.1	500	0
4	0.3	500	0
5	0.5	500	0
6	0.7	500	0
7	1	300	0
8	1	400	0
9	1	500	50
10	2	300	0
11	2	400	0
12	2	500	220
13	3	300	0
14	3	400	0
15	3	500	700
16	3	No	0
17	4	500	400
18	5	500	100
19	3	600	0
20	3	700	0

Systematic experiments that varied Ni loading and the hydrogenation conditions were carried out. Previous studies from our group showed that titania without co-catalyst in MeOH/water is most active for H_2 evolution, when formed at 500 °C for 1 h (this powder shows a 'grey' color) [11–13]. Also in the present work (including Ni decoration and testing for pure water splitting), the most active sample was the one treated at 500 °C for 1 h in H_2. By optimizing the Ni decoration on this grey anatase TiO_2, 3 wt% of Ni loading leads to the most active sample for pure water splitting without the use of any noble metal or sacrificial agent. Moreover, when the hydrogenation temperature was increased to 600 or 700 °C, increasingly black TiO_2 is formed that shows a H_2 evolution rate that decreases to zero. The present findings are in good agreement

with our previous studies, where ‘black’ TiO_2 is not as active as grey TiO_2 due to a different defect distribution. Please see also Figure S1 for more details.

Table S2: Summary of fitting of the femtosecond transient absorption measurement results of representative samples from Figure 3.

Suspensions in neutral H_2O								
sample	λ / nm	a_1	τ_1 / ps	a_2	τ_2 / ps	a_3	τ_3 / ps	$I_0[x10^{-4}]$
**anatase**	450	-0.24		-0.49		-0.27		-3.59
**TiO_2**	650	-0.43	0.92	0.32	15.4	0.25	283	10.60
	1200	0.48		0.25		0.27		0.72
**Grey**	450	-0.32		-0.65		0.13		-8.78
**anatase**	650	-0.64	0.87	0.20	6.4	0.16	170	18.20
**TiO_2**	1200	0.60		0.22		0.18		0.32
**Ni@grey**	450	-0.08		-0.26		0.66		-0.92
**anatase**	650	0.12	0.45	0.45	11	0.43	153	47.30
**TiO_2**	1200	0.34		0.56		0.10		0.15

The time profiles are fitted in a global fit with the lifetimes as global parameters. Three exponential functions were applied for a sufficient fit of the femtosecond time profiles. The sums of the pre-exponential factors were normalized to 1 for better comparison of the relative ratios of the lifetimes. The negative sign of the pre-exponential factor indicates a growth, whereas a positive sign indicates a decay. The equation used to fit the spectra is shown as below:

$$\Delta O.D. = a_1 \exp\left({}^{t - t_0}/_{\tau_1} \right) + a_2 \exp\left(\frac{t - t_0}{\tau_2} \right) + a_3 \exp\left(\frac{t - t_0}{\tau_3} \right) + I_0$$

Table S3: Summary of fitting of the nanosecond transient absorption measurement results of representative samples from Figure 3.

Suspensions in neutral H_2O						
Sample	λ / nm	a_1	τ_1 / ns	a_2	τ_2 / ns	I_0[x10^{-4}]
**anatase TiO_2**	450	-0.98	5.8	0.02	64.3	-0.48
	650	0.05		0.95		-0.55
**Grey anatase TiO_2**	450	-0.91	14	-0.09	820	-0.10
	650	-0.09		0.91		0.25
**Ni@grey anatase TiO_2**	450	-0.13	3210	0.87	14830	-0.42
	650	0.24		0.76		0.11

The time profiles are fitted in a global fit with the lifetimes as global parameters. Two exponential functions were applied for a sufficient fit of the nanosecond time profiles. The sums of the pre-exponential factors were normalized to 1 for better comparison of the relative ratios of the lifetimes. The negative sign of the pre-exponential factor indicates a growth, whereas a positive sign indicates a decay. The equation used to fit the spectra is shown as below:

$$\Delta O.D. = a_1 \exp\left(\frac{t - t_0}{\tau_1}\right) + a_2 \exp\left(\frac{t - t_0}{\tau_2}\right) + I_0$$

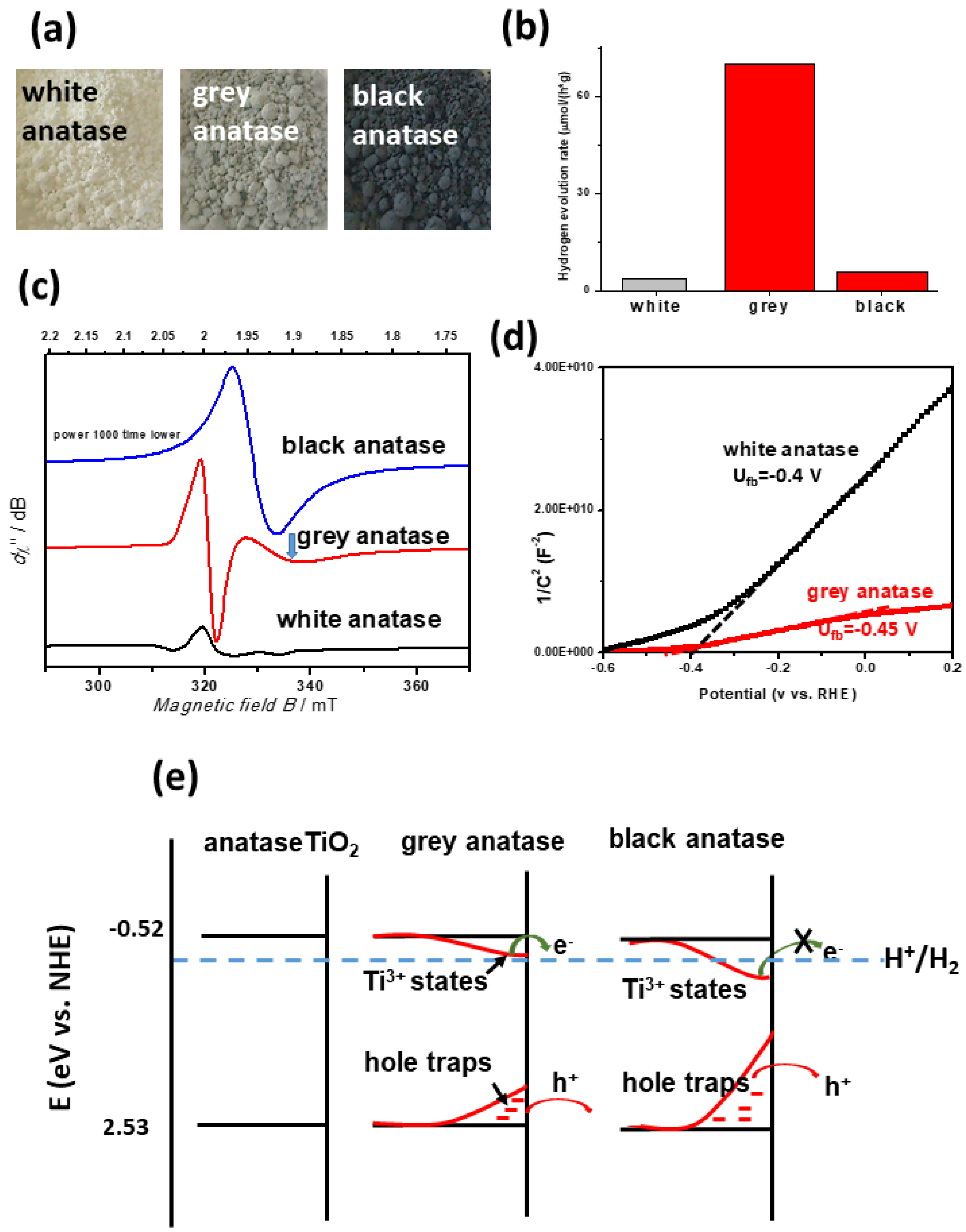

Figure S1 (a) Optical images of unmodified white (commercial anatase, Aldrich), grey and black anatase TiO_2 particles. Grey and black forms were obtained by various hydrogenation treatments (grey anatase: hydrogenation at 500 °C for 1 h; black anatase: hydrogenation at 700 °C for 1 h); (b) photocatalytic hydrogen evolution rate without external co-catalyst under AM 1.5 (100 $mWcm^{-2}$) illumination for different anatase TiO_2 nanoparticles (white, grey and black) in MeOH (50 vol%)/H_2O solution[14]; (c) EPR spectra of anatase TiO_2 nanoparticles

(white, grey and black); (d) Mott-Schottky plots of white and grey anatase sputter deposited layers (1 KHz, 0.1 M Na_2SO_4, pH7) and extraction of the flat band potential (U_{fb}) (resulting donor densities are N_D(white) = 1.11 x 10^{18} cm^{-3} and N_D (grey) = 2.3 x 10^{19} cm^{-3}; (e) Schematic of energy diagram of white, grey and black anatase TiO_2 and relative position of states induced by 'grey' and 'black' treatment. Note band positions in the reference [15] and the red-ox potentials are given for water (at pH 7).

From the figures it is clear that grey anatase has the ability to produce photocatalytic H_2 (i.e. charge transfer to the electrolyte at the level of H^+/H_2).

Clear differences in the defect structure and distribution of states are evident from EPR and time resolved measurements. In line with literature [15], one may deduce from these measurements that the conduction band tailing in grey anatase are more favorable for H_2 generation than in black TiO_2. Also there is a different VB tail (or deep trapping states) for grey and black TiO_2. Regarding the valence band note that the position of the VB deep trapping state in grey is well in line with the PL measurements and the position of the deep trapped holes from time resolved spectroscopy (this is with a favorable exit position for these deep trapped holes for the formation of H_2O_2 (Figure 4b). For grey anatase, the states maximum energy is blue-shifted by approximately 0.5-0.7 eV towards the vacuum level compared with the typical valence band of white anatase, while the valence band maximum for black anatase shifts 1.5 eV towards the vacuum level [16–18]).

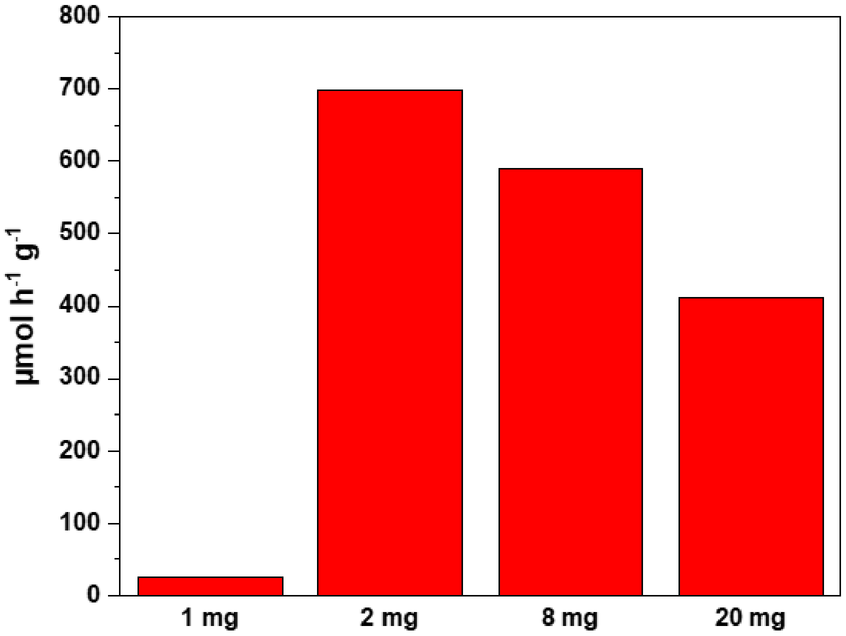

Figure S2 measurements of the H_2 evolution rate for different catalyst loadings of the reactor (schematically shown below).

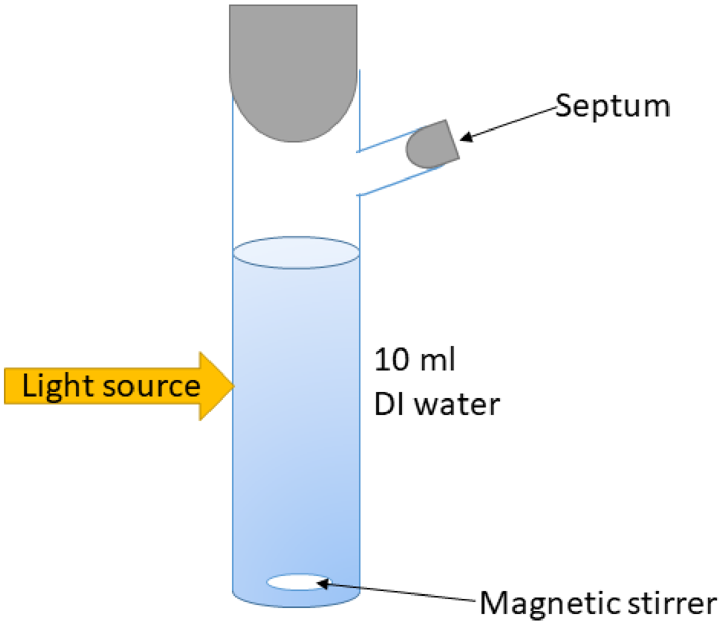

In every case, 10 ml DI water was used with 1 mg, 2 mg, 8 mg and 20 mg TiO_2 based catalyst. The highest activity for our reactor geometry is obtained with 2 mg grey TiO_2.

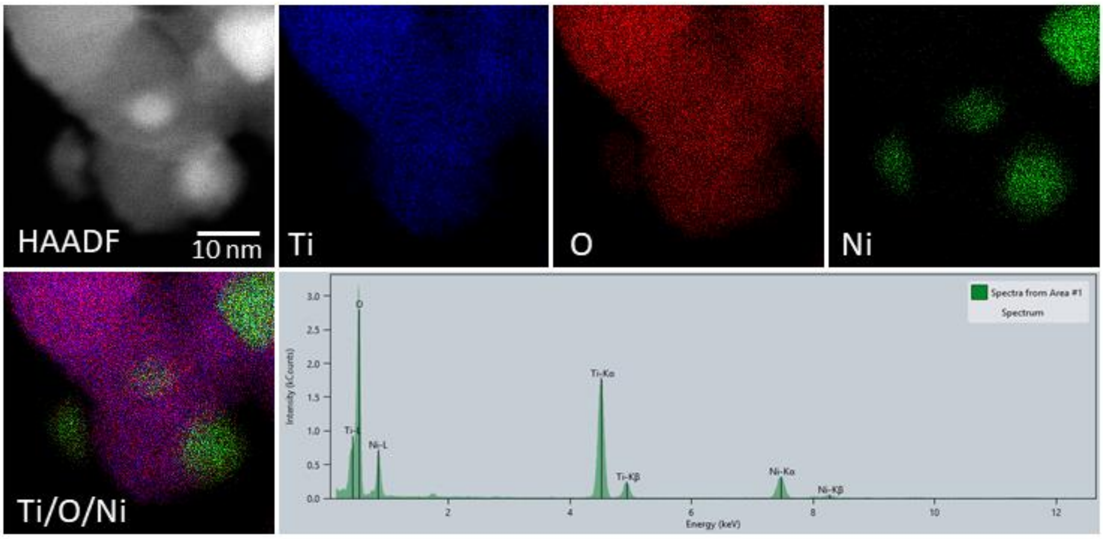

Figure S3 STEM-EDX mapping of Ni@grey anatase TiO_2. EDX mapping of Ti, O and Ni from other representative samples of the type of Figure 1d. After 3 minutes of ultrasonication, the dispersions were drop-casted onto TEM copper grid.

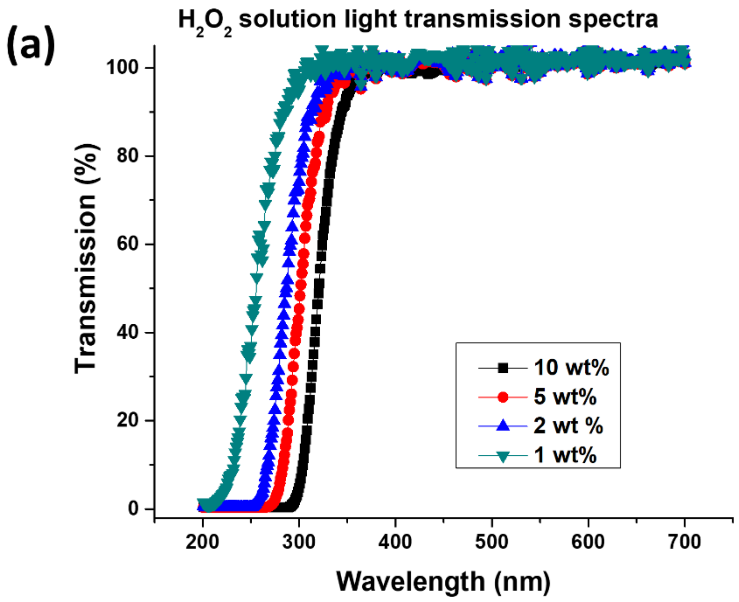

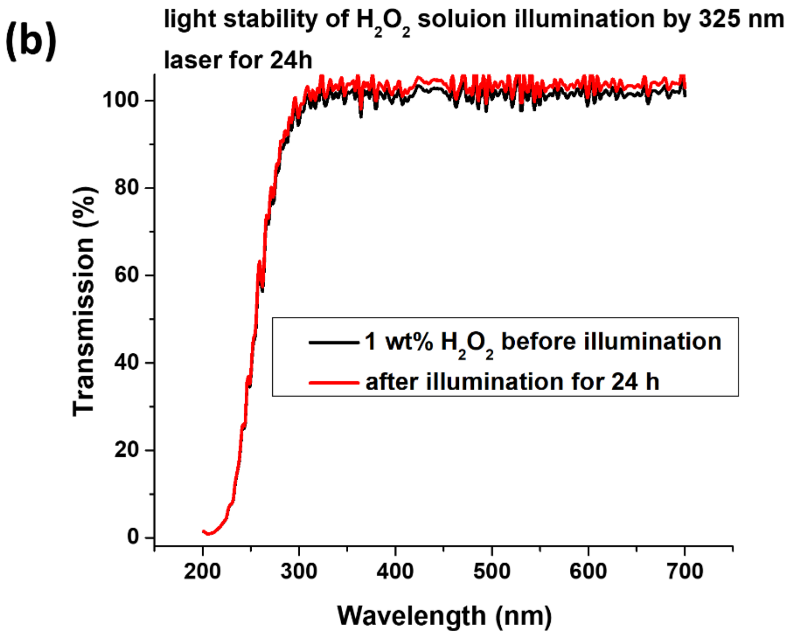

Figure S4 (a) UV-VIS absorption spectra for H_2O_2 solutions with different concentrations; (b) light stability for H_2O_2 solution demonstrated with UV-VIS absorption curves of 1 wt% H_2O_2 solution before and after illumination with 325 nm (60 mW) for 24 h. Under the present experiment conditions no significant light induced decomposition of H_2O_2 takes place (in line with reference [19]).

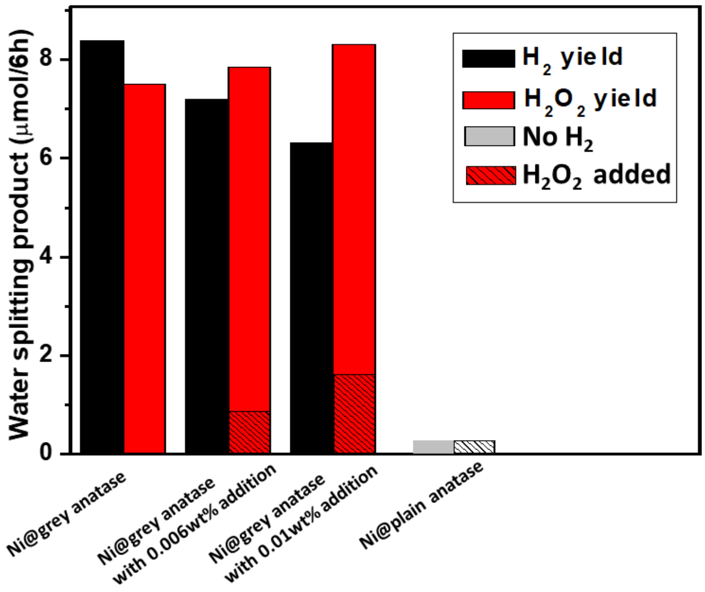

Figure S5 Reference experiments with Ni@plain anatase and with H_2O_2 additions. The experiment demonstrates the inertness of the formed H_2O_2. If H_2O_2 is added in defined amounts to a reacted photocatalytic solution (shaded area) and kept for 24 h, the total H_2O_2 amount remains the same.

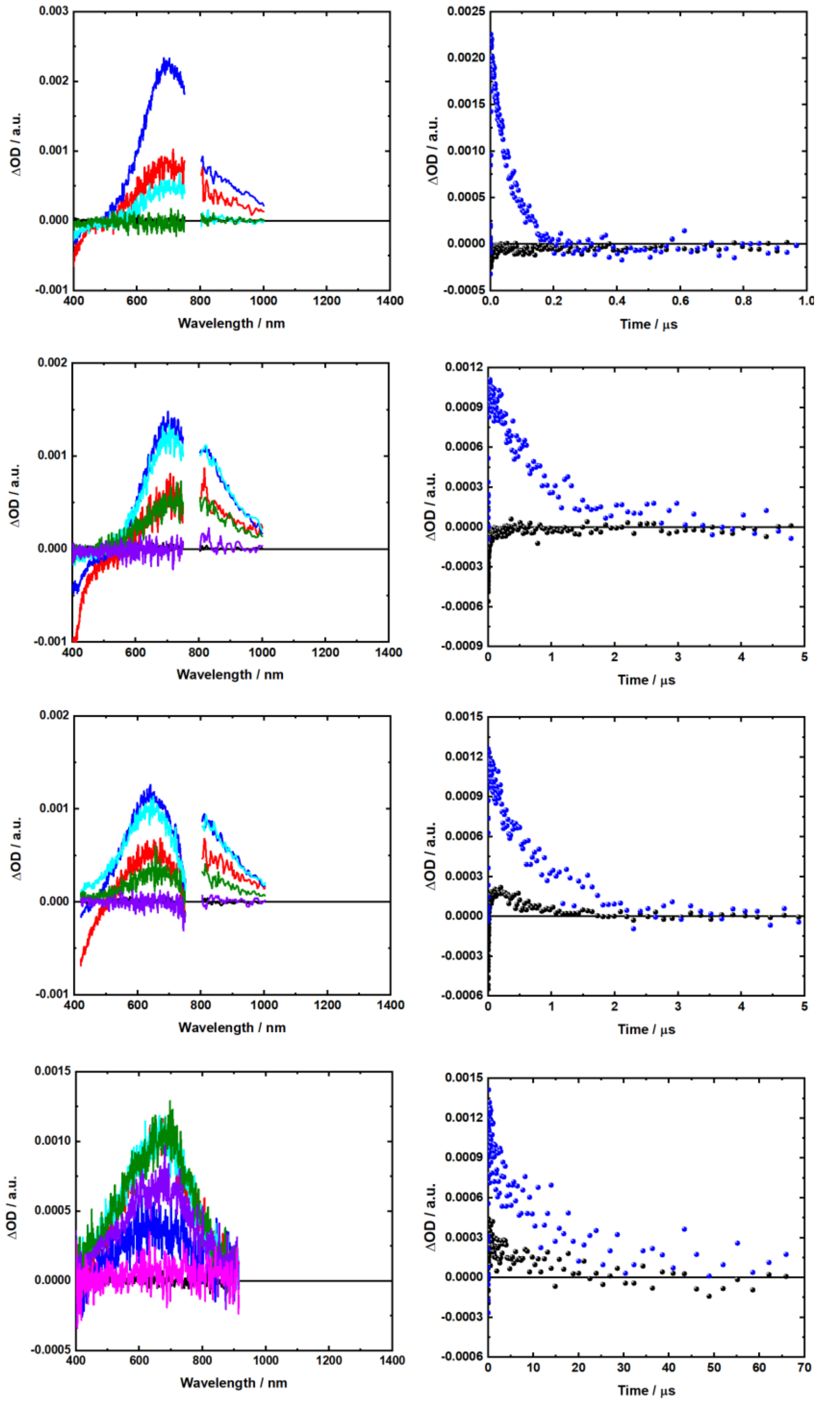

Figure S6 (**Left**) Transient absorption spectra obtained upon nanosecond based laser photolysis (258 nm) of anatase TiO_2, (1st), grey anatase TiO_2, (2nd), partially oxidized Ni@grey anatase TiO_2 (3rd) and fresh Ni@grey anatase TiO_2 (4th from the top) in aqueous argon saturated dispersions at 0 ns (black), 1 ns (red), 10 ns (blue), 100 ns (cyan), 1000 ns (olive), 5000 ns (violet) and 66000 ns (pink). (**Right**) Absorption time profiles of the spectra shown at 450 nm (black) and 650 nm (blue).

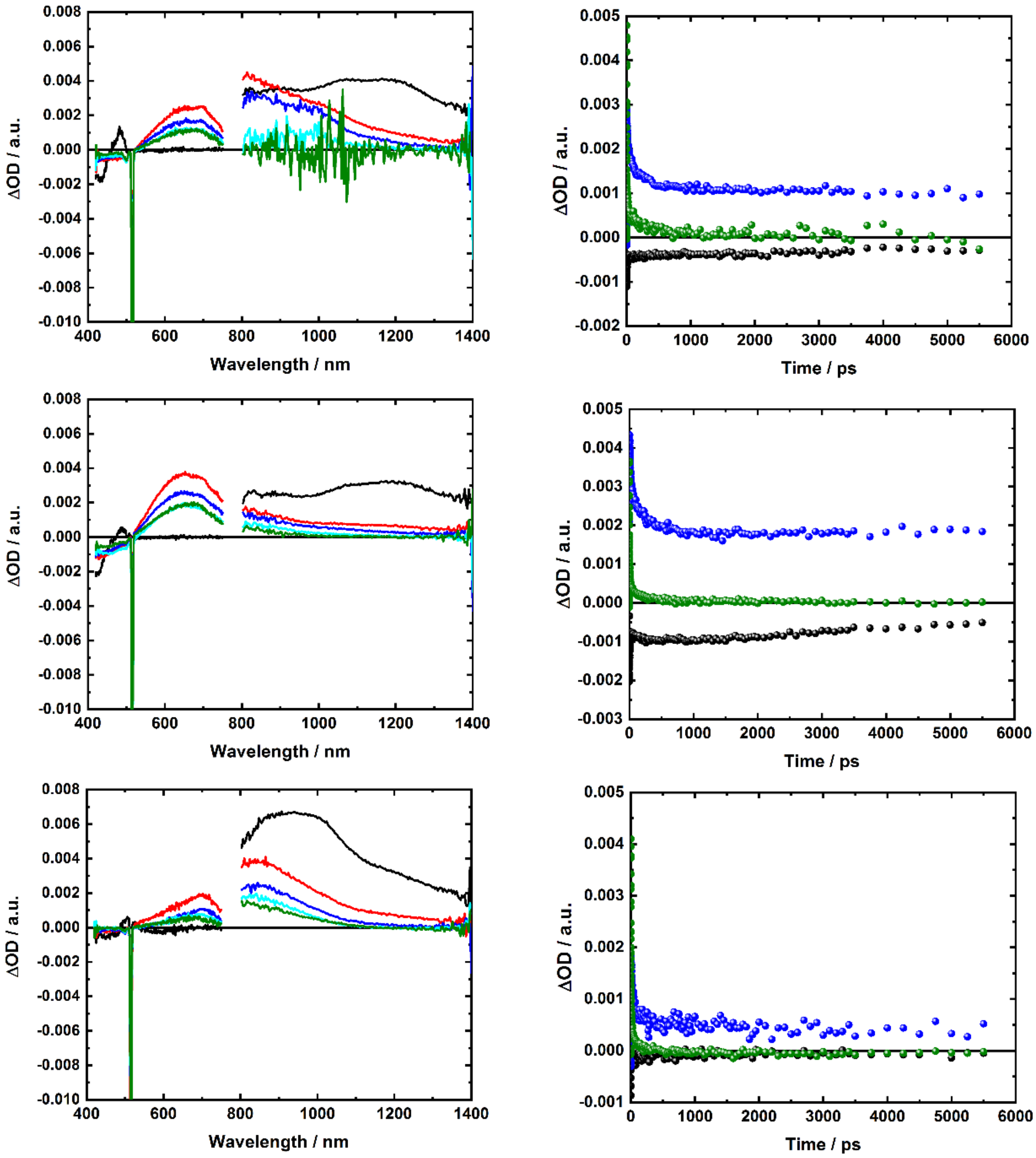

Figure S7 (**Left**) Transient absorption spectra (visible and near-infrared) obtained upon femtosecond based laser photolysis (258 nm) of anatase TiO_2, grey anatase TiO_2, Ni@grey anatase TiO_2 (from the top to the bottom) at 1 ps (black), 10 ps (red), 100 ps (blue), 1000 ps (cyan) and 5500 ps (green). (**Right**) Absorption time profiles of the spectra shown at 450 nm (black), 650 nm (blue) and 1200 nm (green) in aqueous argon saturated dispersions.

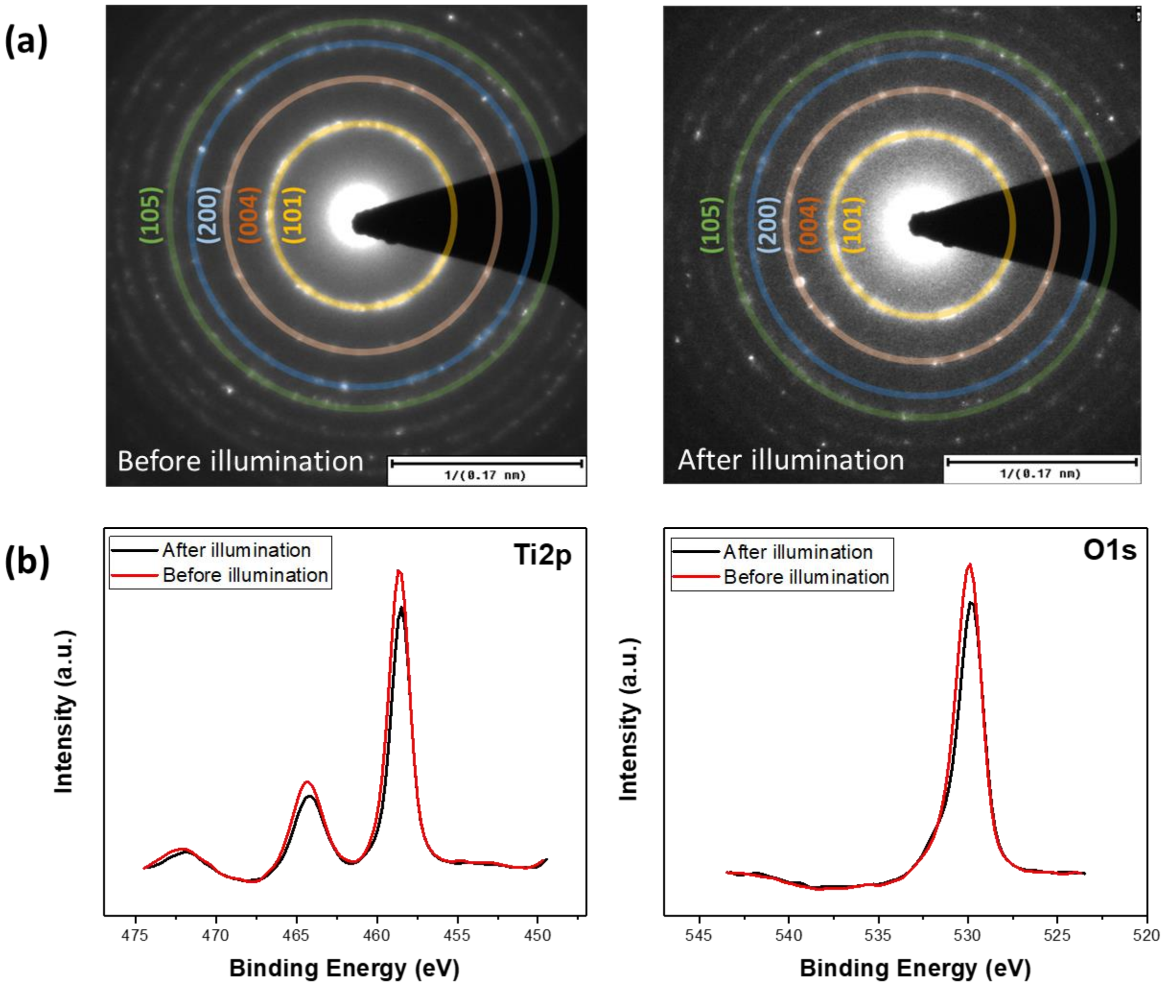

Figure S8 (a) SAED pattern of the Ni@grey anatase before and after UV illumination, (b) comparison of the high-resolution XPS spectra of Ti2p and O1s of the Ni@grey anatase before and after illumination.

References:

[1] G. Demmano, E. Selegny, J.-C. Vincent, Experimental Procedure for a Hydrogen Peroxide Assay Based on the Peroxidase-Oxidase Reaction, Eur. J. Biochem. 238 (1996) 785–789. doi:10.1111/j.1432-1033.1996.0785w.x.

[2] P. Patnaik, J.A. Dean, Dean's analytical chemistry handbook, (2004). http://accessengineeringlibrary.com/browse/deans-analytical-chemistry-handbook-second-edition.

[3] N. V Klassen, D. Marchington, H.C.E. McGowan, H2O2 Determination by the I3- Method and by KMnO4 Titration, Anal. Chem. 66 (1994) 2921–2925. doi:10.1021/ac00090a020.

[4] P. Salvador, F. Decker, The generation of hydrogen peroxide during water photoelectrolysis at n-titanium dioxide, J. Phys. Chem. 88 (1984) 6116–6120. doi:10.1021/j150669a013.

[5] Y. Nosaka, A.Y. Nosaka, Generation and Detection of Reactive Oxygen Species in Photocatalysis, Chem. Rev. 117 (2017) 11302–11336. doi:10.1021/acs.chemrev.7b00161.

[6] J.R. Harbour, J. Tromp, M.L. Hair, Photogeneration of hydrogen peroxide in aqueous TiO2 dispersions, Can. J. Chem. 63 (1985) 204–208. doi:10.1139/v85-032.

[7] C. Kormann, D.W. Bahnemann, M.R. Hoffmann, Photocatalytic production of hydrogen peroxides and organic peroxides in aqueous suspensions of titanium dioxide, zinc oxide, and desert sand, Environ. Sci. Technol. 22 (1988) 798–806. doi:10.1021/es00172a009.

[8] T. Liu, X. Li, X. Yuan, Y. Wang, F. Li, Enhanced visible-light photocatalytic activity of a TiO2 hydrosol assisted by H2O2: Surface complexation and kinetic modeling, J. Mol. Catal. A Chem. 414 (2016) 122–129. doi:https://doi.org/10.1016/j.molcata.2016.01.011.

[9] S. Na-Phattalung, M.F. Smith, K. Kim, M.-H. Du, S.-H. Wei, S.B. Zhang, S. Limpijumnong, First-principles study of native defects in anatase $\backslashmathrm{\{}Ti{\}}{\backslashmathrm{\{}O{\}}}_{2}$, Phys. Rev. B. 73 (2006) 125205. doi:10.1103/PhysRevB.73.125205.

[10] A.H. Boonstra, C.A.H.A. Mutsaers, Adsorption of hydrogen peroxide on the surface of titanium dioxide, J. Phys. Chem. 79 (1975) 1940–1943. doi:10.1021/j100585a011.

[11] N. Liu, C. Schneider, D. Freitag, U. Venkatesan, V.R.R. Marthala, M. Hartmann, B. Winter, E. Spiecker, A. Osvet, E.M. Zolnhofer, K. Meyer, T. Nakajima, X. Zhou, P. Schmuki, Hydrogenated Anatase : Strong Photocatalytic Dihydrogen Evolution without the Use of a Co-Catalyst ** Angewandte, (2014) 14425–14429. doi:10.1002/ange.201408493.

[12] N. Liu, C. Schneider, D. Freitag, M. Hartmann, U. Venkatesan, J. Müller, E. Spiecker, P. Schmuki, Black TiO2 Nanotubes: Cocatalyst-Free Open-Circuit Hydrogen Generation, Nano Lett. 14 (2014) 3309–3313. doi:10.1021/nl500710j.

[13] N. Liu, C. Schneider, D. Freitag, E.M. Zolnhofer, K. Meyer, P. Schmuki, Noble-Metal-Free Photocatalytic H2 Generation: Active and Inactive 'Black' TiO2 Nanotubes and

Synergistic Effects, Chem. - A Eur. J. 22 (2016) 13810–13814. doi:10.1002/chem.201602714.

[14] N. Liu, X. Zhou, N.T. Nguyen, K. Peters, F. Zoller, I. Hwang, C. Schneider, M.E. Miehlich, D. Freitag, K. Meyer, D. Fattakhova-Rohlfing, P. Schmuki, Black Magic in Gray Titania: Noble-Metal-Free Photocatalytic H2 Evolution from Hydrogenated Anatase, ChemSusChem. 10 (2017) 62–67. doi:10.1002/cssc.201601264.

[15] J. Xue, X. Zhu, Y. Zhang, W. Wang, W. Xie, J. Zhou, J. Bao, Y. Luo, X. Gao, Y. Wang, L. Jang, S. Sun, C. Gao, Cover Picture: Nature of Conduction Band Tailing in Hydrogenated Titanium Dioxide for Photocatalytic Hydrogen Evolution (ChemCatChem 12/2016), ChemCatChem. 8 (2016) 1991–1991. doi:10.1002/cctc.201600682.

[16] A. Naldoni, M. Allieta, S. Santangelo, M. Marelli, F. Fabbri, S. Cappelli, C.L. Bianchi, R. Psaro, V. Dal Santo, Effect of Nature and Location of Defects on Bandgap Narrowing in Black TiO2 Nanoparticles, J. Am. Chem. Soc. 134 (2012) 7600–7603. doi:10.1021/ja3012676.

[17] X. Chen, L. Liu, P.Y. Yu, S.S. Mao, Increasing Solar Absorption for Photocatalysis with Black Hydrogenated Titanium Dioxide Nanocrystals, Science (80-.). 331 (2011) 746–750. http://science.sciencemag.org/content/331/6018/746.abstract.

[18] M.E. Khan, M.M. Khan, B.-K. Min, M.H. Cho, Microbial fuel cell assisted band gap narrowed TiO2 for visible light-induced photocatalytic activities and power generation, Sci. Rep. 8 (2018) 1723. doi:10.1038/s41598-018-19617-2.

[19] S. Goldstein, D. Aschengrau, Y. Diamant, J. Rabani, Photolysis of Aqueous H2O2: Quantum Yield and Applications for Polychromatic UV Actinometry in Photoreactors, Environ. Sci. Technol. 41 (2007) 7486–7490. doi:10.1021/es071379t.
